# Platelet-rich plasma therapy in erectile dysfunction and Peyronie’s disease: a systematic review of the literature

**DOI:** 10.1007/s00345-024-05065-3

**Published:** 2024-05-29

**Authors:** Maria Giovanna Asmundo, Emil Durukan, Elena von Rohden, Sandra Amalie Thy, Christian Fuglesang Skjødt Jensen, Mikkel Fode

**Affiliations:** 1 Urology Section, University of Catania, Catania, Italy; 2 Department of Urology, Copenhagen University Hospital – Herlev and Gentofte, University of Copenhagen, Herlev, Denmark; 3 Department of Clinical Medicine, University of Copenhagen, Copenhagen, Denmark

**Keywords:** Andrology, Erectile dysfunction, Male infertility, Peyronie’s disease, Platelet-rich plasma

## Abstract

**Purpose:**

Platelet-rich plasma (PRP) as a regenerative therapy has gained interest in the field of andrology for the treatment of erectile dysfunction (ED) and Peyronie’s disease (PD). This systematic review aims to critically evaluate the current evidence on the use of PRP for these conditions.

**Methods:**

We performed a systematic literature search according to the PRISMA guidelines using PubMed and Scopus databases in December 2023. Studies were included if they evaluated the effect of PRP therapy for ED or PD in humans.

**Results:**

We identified 164 articles, 17 of which were included, consisting of 11 studies on ED, 5 studies on PD, and 1 study on both. We included four randomized controlled trials, 11 prospective cohort studies, and three retrospective cohort studies including a total of 1099 patients. The studies on ED and PD generally showed small to moderate benefits with mild and transient side effects and no major adverse events were reported. General limitations included variations in PRP protocols, small sample sizes, short follow-up periods, and lack of control groups except in the three randomized trials on ED and the one on PD.

**Conclusion:**

The literature on PRP therapy in andrology is limited and difficult to interpret due to variations in protocols and methodological drawbacks. Further research is necessary to determine the optimal preparation and treatment protocols for PRP therapy and clarify its effectiveness in andrology.

**Supplementary Information:**

The online version contains supplementary material available at 10.1007/s00345-024-05065-3.

## Introduction

Platelet-rich plasma (PRP) refers to the liquid fraction of peripheral blood, which has been processed to ensure a high concentration of platelets [1]. Platelets have a crucial role in the aggregation process and promote coagulation through adhesion, activation, and aggregation processes [2]. However, recent studies revealed a broader perspective on platelets and their functions. Since the platelets are rich in growth factors (GF), PRP preparations are believed to have potential in regenerative medicine [3]. The main GFs released by platelets in an inflammatory environment are platelet-derived GF (PDGF), fibroblast GF (FGF), epidermal GF (EGF), insulin-like GF (IGF) as well some interleukin (IL) [4]. In PRP therapy, a blood sample is collected and centrifuged to isolate the relevant fraction. This is then injected into the tissue where an effect is desired. PRP therapy has been used in various conditions, such as musculoskeletal injuries, wound healing, and dermatological disorders [1]. PRP appeared to demonstrate mitogenic, chemotactic, and angiogenic properties, rooted in its ability to induce soft tissue proliferation and collagen deposition by activating fibroblasts [5]. Recently, PRP therapy has also gained increasing interest in the field of andrology for the management of erectile dysfunction (ED) and Peyronie's disease (PD) [6]. According to the literature, PRP has demonstrated neurotrophic effects on damaged nerves. Animal studies focusing on erectile function in male rats with cavernous nerve injuries have reported improved erections following PRP therapy compared to the injured control group [7, 8]. Notably, PRP may enhance axon myelination, reduce apoptosis, and facilitate fiber regeneration. However, due to a paucity of robust clinical studies, there are no official recommendations for the use of PRP in these conditions and the guidelines of the European Association of Urology (EAU) or American Association of Urology (AUA) specifically denotes it as experimental in ED and PD [9]. In this systematic review, we aim to provide an overview of the current evidence on the use of PRP therapy in the management of ED and PD. We will review the available clinical studies, discuss the potential mechanisms of action of PRP, and highlight the limitations and future directions of this therapy in andrology.

## Methods

### Evidence acquisition

We registered the protocol in the PROSPERO database (ID CRD42024495624) and reported according to the PRISMA guidelines [10].

### Search strategy

Two authors (M.G.A. and E.D.) conducted a comprehensive bibliographic search on MEDLINE and Scopus on January 16th, 2024 to identify studies published since 1995 describing the benefits of PRP therapy in the management of ED and PD. The following search strings were used:

- Erectile Dysfunction: ("erectile dysfunction" OR "sexual dysfunction" OR "impotence") AND ("platelet rich plasma" OR "PRP")
- Peyronie’s Disease: (“peyronie’s disease” OR “penile curvature” OR “penile induration”) AND (“platelet rich plasma” OR “PRP”)
- (TITLE-ABS-KEY (erectile dysfunction OR sexual dysfunction OR impotence) AND TITLE-ABS-KEY (platelet rich plasma OR PRP) AND (LIMIT-TO (DOCTYPE, "ar")))
- (TITLE-ABS-KEY ((peyronie’s disease OR penile curvature OR penile induration) AND TITLE-ABS-KEY (platelet rich plasma OR PRP) AND (LIMIT-TO (DOCTYPE, "ar")))

### Study selection

We used the Population, Intervention, Comparator, Outcome, Study (PICOS) model to define study eligibility [11]. PICOS criteria were set as follows: Population—patients affected by ED or PD; Intervention—autologous PRP injection; Comparator—Human patients affected by ED or PD receiving other types of treatments or no treatments at all; Outcome—variations of degree of penile curvature or erectile function in terms of IIEF, IIEF-5, IIEF-EF, ED duration, Erection Hardness Score [EHS], SEP [Sexual Encounter Profile], end-diastolic velocity [EDV], peak systolic velocity [PSV], resistive index [RI] or arterial diameter. Study—retrospective and prospective studies (Supplementary Table 1). No minimum number on patient population was applied.

Only English-language articles were considered for inclusion, with case reports, review articles, and publications with missing full texts (abstracts only) being excluded. Only studies conducted on human patients were included. Additional references were sought by hand-searching the reference lists of included studies and identified review papers. Due to recognized discrepancies in methodology, the decision was made to describe individual studies without conducting meta-analyses.

### Data extraction

We recorded the following items: first author, study design, sample size, baseline parameters and post-treatment parameters (patients’ age, IIEF, IIEF 5, IIEF-EF, degree of curvature, ED duration, EHS, SEP, EDV, PSV, [RI or arterial diameter], type of treatment, treatment dose and number of doses. Discrepancies in study selection were resolved by consensus with the coauthors. Statistical analyses were not performed.

### Risk of bias

Two authors (M.G.A and E.D.) assessed the risk of bias and discrepancies were resolved by consensus with the coauthors. We employed the Cochrane Collaboration's Risk of Bias tool to evaluate the risk of bias in randomized trials [12]. This involved assessing criteria such as random sequence generation, allocation concealment, blinding of participants and personnel, blinding of outcome assessment, incomplete outcome data, selective reporting, and other potential biases. In the case of comparative non-randomized studies, we utilized the Risk of Bias in Non-randomized Studies of Interventions (ROBINS-I) tool, evaluating criteria such as confounding, participant selection, intervention measurement, deviations from intended interventions, missing data, outcome measurement, selection of reported results, and overall risk of bias [13]. For single-arm studies, we evaluated the risk of bias using the criteria recommended by the European Association of Urology Guidelines Office, covering aspects like a priori protocol, participant selection, adequate handling of missing data, specification of outcomes/selective reporting, and measurement of outcomes.

## Evidence synthesis

### Description of the studies included

**Fig. 1:**
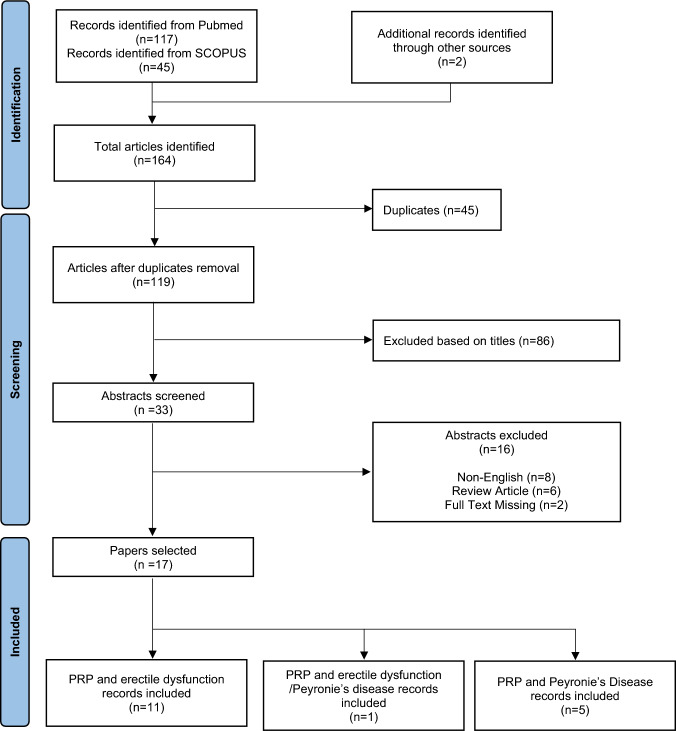
Flow diagram of literature search and study selection. *Consider, if feasible to do so, reporting the number of records identified from each database or register searched (rather than the total number across all databases/registers). **If automation tools were used, indicate how many records were excluded by a human and how many were excluded by automation tools.From: Page MJ, McKenzie JE, Bossuyt PM, Boutron I, Hoffmann TC, Mulrow CD, et al. The PRISMA 2020 statement: an updated guideline for reporting systematic reviews. BMJ 2021;372:n71. https://doi.org/10.1136/bmj.n71. For more information, visit: http://www.prisma-statement.org/

A total of 164 records were identified. After duplicates removal, 119 papers were subdued to titles screening. The remaining 33 abstracts were screened, and after excluding 16 records, a total of 17 papers were deemed eligible for review (Fig. 1). All clinical studies used autologous PRP but both preparation and injection methods differed across studies as specified in Tables 1 and 2.

**Table 1:** Studies investigating Platelet-rich Plasma therapy in men with Erectile Dysfunction

Author	Year	Study design	Study population	Inclusion/ Exclusion Criteria	Groups	PRP preparation method	Treatment method	Results	Adverse effects	Limitations
Matz [20]	2018	Retrospective Cohort Study	5 patients with organic ED (of which 1 with both ED + PD, mean age: 45 years (IQR: 27–61)	Inclusion criteria: Vasculogenic ED, penile fracture, medication-related and electrical injury to the genitalia Exclusion criteria: not reported	N/A	Autologous PRFM (PRP activated by calcium chloride)	Between 4 and 9 mL of PRFM injected per treatment session. Intracavernosal injection was performed for ED. Follow up: 15.5 months	IIEF-5 scores improved by an average of 4.14 points after PRFM therapy	In all patients: 4 with mild pain, 1 experienced bruising. No major adverse events	Small sample size, no control group. PRP injections were given randomly (multiple times if requested)
Geyik [21]	2021	Retrospective Cohort Study	Group 1: 93 patients Mean age (SD): 51.23 (± 11.36) years Group 2: 91 patients Mean age (SD): 46.9 (± 11.89) years	Inclusion criteria: Vasculogenic ED Patients with < 26 IIEF-EF score after use of daily 5 mg of tadalafil for at least 3 months Exclusion criteria: glycated haemoglobin > 7 ng/ml; hypogonadism (testosterone < 4 ng/ml); non-adjusted cardiac and antihypertensive medications with consultations; pelvic surgery; degenerative neurological disorders, not followed up; data unavailability	Group 1: Li-SWT, Group 2: Li-SWT with PRP	Autologous PRP self-gelled and citrated (Ycellbio PRP). Sample was centrifuged at 3.700 (RPM)	Each PRP injection (3–4 ml) was administered 10–14 days apart three times in one course. 1 intracavernosal and 3 subcutaneous areas (both right and left lateral neural lines and dorsal balanic submucosal region	Group 1: IIEF-EF (Baseline: 14.33 ± 4.39, 6 months: 23.8 ± 4.37 (*p* < 0.001); Group 2 IIEF-EF (Baseline 17.82 ± 3.44, 6 months 26.3 ± 2.55 (*p* < 0.001). No difference between groups	All patients in Group 2 reported a temporary pain at the injection site, and 24 (26.4%) of them had mild penile bruising after the injection despite the absence of bleeding diathesis	Retrospective design. PRP administered with Li-SWT. No control group
Poulios [26]	2021	Prospective, randomized, double-blind, placebo-controlled trial	PRP group: 30 patients, median age: 58 years (IQR: 51.5–62) Placebo group: 30 patients, median age: 59 years (IQR: 53.5–61)	Inclusion criteria: Sexually active men aged 40–70 years old in stable, heterosexual relationship (> 3 months); vasculogenic ED; no PDE-5i 1 month before treatment and IIEF-EF score 11–25; no PDE-5i during the study; regular sexual intercourse (4 times/month) without influence of alcohol or drug and documented using SEP Exlclusion criteria: major pelvic surgery/trauma; major penile surgery/radiation; priapism; penile fracture; Peyronie’s disease; abnormal testosterone (< 300 ng/dL or > 1197 ng/dL); pshycogenic ED; severe pshychiatric condition; partner’s sexual dysfunction or pregnant/breastfeeding	PRP group and Saline group. All: Presence of mild or moderate ED after washout from PDE5i or other treatment	Autologous PRP. Processed by Magellan Autologous Platelet Separator	Tourniquet clipped around the base of penis and removed 20 min after the procedure. A total of 5 ml was infused in each corpus cavernosum over a 2-min period. PRP group: 2 injections of PRP with one-month interval. Placebo group: 2 injections with saline with one-month interval	1 month: PRP group: MCID in 22/29 (76%); Placebo group: MCID in 7/28 (25%) (*p* < 0.001), 3 months: PRP group: MCID in 20/29 (69%); Placebo group: 10/26 (39%) (*p* = 0.018), 6 months: PRP group MCID in 20/29 (69%). Placebo group: 7/26 (27%) (*p* < 0.001)	VAS score higher in placebo group compared to PRP (2.6 ± 0.4 vs 2.2 ± 0.6, respectively, *p* = 0.008), no transient hemorrhagic adverse events (hematuria, local petechial bleeding or ecchymosis) or other side effects reported	Small sample size, short follow-up, no inclusion of severe ED, did not evaluate the qualitative or quantitative composition of growth factors, cytokines or other molecules
Taş [19]	2021	Prospective Cohort Study	31 patients Mean age (SD) 54.41 (± 8.74) years	Inclusion criteria: Vasculogenic ED with metabolic syndrome and no previous ED treatment Exclusion criteria: patients with neurological deficits who had previously received ED treatments and did not agree to participate in the study	N/A	Autologous PRP. Sample was centrifugated at 2.800 rpm for 8 min. A solution was prepared containing 1.000–2.000 × 10^3/uL PRP	3 ml PRP was injected into each corpora cavernosa with a 25-gauge needle (total amount = 3 * (1.000–2.000) * 10^6. During intracavernosal injection, clamping (20 min) was performed with Stockmann penis clamp. PRP was applied 3 times at an interval of 15 d. The injection sites vary by 1 cm in the mid-penile region	19 patients (61.29%) improved after 6 months. Preprocedural IIEF-EF values of the patients were significantly lower than the 1, 3, and 6 months after the procedure (*p* < 0.001)	No patients experienced pain during the procedure; slight subcutaneous bruising occurred after only 8 (8/93) applications at the injection site. In the first follow-up of a patient after the 3rd injection, a 4-mm diameter fibrotic plaque was observed on the ventral side in the middle of the penis shaft. Not noticed by the patient and did not cause pain, shortening of the penis, thinning, or curvature	Small sample size, no randomization, no control group, short follow up period
Wong [22]	2021	Prospective Cohort Study	29 patients Mean age (SD): 54.93 (± 8.31) years	Inclusion criteria: heterosexual men 30–75 yeard old diagnosed with ED > 6 months and who did not respond to conservative treatments Exclusion criteria: previous androgen deprivation therapy, history of penile surgery or penile disorders, such as priapism or Peyronie’s disease, or had a severe systemic disease or local skin infection	N/A	Autologous PRP. Sample centrifugated at 500G and 1500 G for 15 min	Equal volumes of 1–2 mL PRP were injected into each corpus cavernosus through a 27-gauge needle in accordance with a sterile protocol A penile injection of PRP every 3 weeks for 3 times	Mean IIEF-5 score pretreatment was 12.034 (± 5.10) and posttreatment: 16.59 (± 5.5) (*p* < 0.001)	Two participants reported a small induration at the injection site after the first injection, but both also reported no hindrances in sexual intercourse	Small sample size, no randomization, no control group, short follow up period
Sajjad [23]	2021	Prospective, non-randomized, Cohort Study	60 patients Group A: 30 patients mean age (SD) 42.56 (± 7.44) years; Group B: 30 patients mean age (SD) 45.89 (± 9.11) years	Inclusion criteria: pathological ED	Group A: Li-SWT Group PRP	Autologous PRP with calcium chloride solution. Sample was centrifugated to 500 G for 5 min and 1500 G for 3 min	Injection of 1 mL of PRP on the lateral surface of the penis distally and 1 mL proximally; 1 mL approaching albuginea; 0.5 mL in each ischia-cavernous muscle; 1 mL in each peduncle of the penis. Each injection was repeated weekly for 6 weeks LiSWT protocol comprised 300 shocks in 5 anatomical sites of the penis during a 3-min session twice per week for 3 weeks; patients received 2 sessions after a 3-weeks interval Follow up: 3 months	The mean (SD) score in IIEF-5 for LESWT and PRP was 20.21 and 21.26 at 12th week (*p* > 0.05) with no statistically significant difference. The EHS and SEAR improvement after 12 weeks was not statistically significant	Two participants reported local transient penile pain	Small sample size; no randomization; no placebo group; short follow up period
Zaghloul [18]	2021	Prospective Cohort Study	34 patients Mean age (SD): 50.18 (± 8.64) years	Inclusion criteria: ED for > 6 months not responding to oral on-demand or daily PDE-51; regular sexual intercourses (1 per week) Exclusion criteria: Peyronie’s disease; use of drugs that affect erectile dysfunction (i.e. psychotropic medications); psychogenic disorders;	Not applicable: all patients received oral daily dose of 5 mg tadalafil with oral on-demand vardenafil 20 mg (included only non-responders)	Autologous PRP without activation factors. Sample was centrifugated at 1000 rpm for 5 min, then at 3000 rpm for 5 min	Injection of 0.5 ml of PRP concentrate in each corpus cavernosum each week for 2 months using an 8 mm 28-gauge needle. Pressure applied on the root of the penis for 1–2 min and massage of the penis for 1–2 min to distribute the PRP. 3 months follow-up	Mean ED duration: 26.5 ± 23.83 months, IIEF-5 score change -5.5, ± 5.2, *p* = < 0.001 (before 7.7059 ± 2.73617, after 13.2059 ± 6.77240), no change in EDV, PSV, RI or arterial diameter (assessed using 20 µg PGE1)	None reported	Small sample size, no randomization, no control group, short follow-up
Zaghloul [18]	2022	Prospective Cohort Study	Group 1: 24 patients with ED and diabetes, mean age (SD) 50.16 (± 6.89) years, Group 2: 24 non-diabetic ED patients, mean age (SD): 52.33 (± 5.17) years	Inclusion criteria: ED patients not responding to on demand PDE-5i Exclusion criteria: Peyronie’s disease; psychotropic medications; major tranquillizers; smoking status	Diabetic/non-diabetic ED patients, all patients received oral daily dose of 5 mg tadalafil with oral on-demand vardenafil 20 mg (included only non-responders) and PRP	Autologous PRP	PRP injected with 7-9 ml PRP in total. The patients were injected 3 times at monthly intervals. They used an 8 mm 28-gauge needle, injecting at 4 injection points, 2 on each side of the penis (one in the proximal third and one in the middle third). Pressure applied on the root of the penis for 1–2 min and massage of the penis for 1–2 min to distribute the PRP	Improvement of mean total IIEF-5 scores in the diabetic group (8.04 vs. 12.1, *p* = 0.003) as well as in the non-diabetic group (10.2 vs. 14.8, *p* = 0.001), improved response to on-demand 20 mg vardenafil at 3 months follow-up assessed by EHS scores, improvement of pharmaco-penile duplex (increase in mean PSV, decrease in mean EDV)	Mild pricking pain during injections	Small sample size, no randomization, no control group, short follow-up
Schirmann [24]	2022	Prospective cohort study	15 patients, mean age (IQR), 55 (49–64) years	Inclusion criteria: Moderate and severe vasculogenic ED who had filed 1st or 2nd lines of treatment; not surgery treatment Exclusion criteria: androgenic deficiency	N/A	Autologous PRP. Sample was centrifugated for 3 min at 3000 rpm	3 mL of PRP were injected in each corpus cavernosum plus 6 mL were injected subcutaneously at each session. All patients received 3 injections 15 days apart	Improvement in IIEF-EF score: baseline 11.80 (± 5.51), 1 month 16.80 (± 4.97; *p* = 0.001), 3 months 16.23 (± 5.10; *p* = 0.003), 6 months 15.15 (± 6.44; *p* = 0.02). Sexually discomfort score was only improved after 1 month (6.67 ± 19.97 vs 16.67 ± 24.40; *p* = 0.043) EHS and SEP score were not improved	None reported	Small sample size; no randomization; no placebo group; short follow-up
Shaher [16]	2023	Prospective randomized, double-blind, placebo-controlled trial	PRP group: 55 patients, median age 56 years Placebo group: 54 patients, median age 54 years	Inclusion criteria: Sexually active men aged 45–65; mild/mild-moderate/moderate ED; no ED treatments. Vasculogenic ED Exclusion criteria: major pelvic surgery or trauma; major penile surgery or radiation; anatomical or pathological disorders impairing penile erection; low testosterone (< 300 ng/dL); thrombocytopenia; psychogenic ED	PRP group and saline (placebo) group	Autologous PRP	3 mL injected into each corpora cavernosa at 3 different sites: 1 cm proximal to the corona, 1 cm distal to the root of the penis, and at the mid-penile shaft. Placebo group: Saline (3 mL) at the same sites. Procedure was repeated twice at 2-week intervals	1 month: PRP group: 38 (76%), Placebo group: 9 (18%) (*p* < 0.001); 3 months: PRP group 36 (72%), Placebo group: 8 (16%) (*p* < 0.001); 6 months: 35 (70%), Placebo group 8 (16%) (*p* < 0.001)	No difference in VAS between the PRP and saline groups (1.52 ± 1.2 vs 1.54 ± 1.3, respectively). No complications were reported	No severe ED in the study population, short follow-up period
Francomano [25]	2023	Prospective single arm, non-randomized	150 patients with vasculogenic ED, non-responders to PDE-5i, mean age (SD) 51 (± 16.7) years	Inclusion criteria: Sexually active male patients > 18 years-old; not responders to PDE-5i during the previous 3 months; normal blood platelet number, hormonal profile; IIEF-5 between 6–21 and PSV < 35 cm/s; no ED treatments during the study; regular sexual intercourse (4 times/month) Exclusion criteria: Non-nerve sparing RP; not controlled metabolic diseases; priapism; low testosterone; high PSA; psychogenic ED; pelvic surgery or RT; pshychiatric condition	N/A	Autologous PRP. Sample was centrifugated at 1500 rpm for 15 min	5 mL of PRP injected into each corpora cavernosa at two different lateral sites, 1 cm distal to mid penile shaft through a 25 Gauge needle. A rubber band was applied around the penile root and removed 20 min after the injections Follow up time: 1 month	Improvement of IIEF-5 score questionnaire (12 ± 2.6 vs. 19 ± 3.0; *p* < 0.0001) and PSV (32 ± 5.5 cm/s vs. 42 ± 7.6 cm/s; *p* < 0.0001) Mean platelet volume (MPV) has a significant accuracy in identifying men clinically responding to PRP with favorable outcomes	16 subjects experienced dull pain during injections and two patients reported a slight subcutaneous hematoma at the injection site the day after the procedure	No randomized trial; no control group; single injection; short follow up
Masterson [27]	2023	Prospective, randomized, double-blind, placebo-controlled trial	61 patients PRP group: 28 patients, median age 49 years ([IQR] 38.5–55). placebo group: 33 patients median age 46 years ([IQR] 42–56)	Inclusion criteria: male patients aged 30–75 with organic ED (IIEF score 11–25) for > 6 months; normal testosterone; hemoglobin A1c < 9% Exclusion criteria: Patients using intracavernosal injection or urethral suppositories for ED treatment;	PRP and saline solution group	Autologous PRP analyzed by Arthrex Angel PRP system	2.5 mL of PRP, or saline solution, were injected into the right and left corpus cavernosum at each of the two session that were scheduled 28 ± 7 days apart. Application of a torniquet at the base of the penis and removal 20 min after injection	MCID at 1 months 14 (58.3%) in platelet-rich plasma vs 15 (53.6%) in placebo (*p* = 0.7) IIEF-EF changed from 17.4 (15.8–19.0) to 21 (17.9–24.0) at 1 month in PRP group (*p* = 0.02), vs 18.6 (17.3–19.8) to 21.6 (19.1–24.1) in the placebo group (*p* = 0.009). Mean changes from baseline at 3 months were not significant; mean changes from baseline at 6 months were statistically significant in both groups (mean increases of 5, [IQR] 1.9–8.1, *p* = 0.003 in PRP vs 2.2 [IQR] 0.1–4.3; *p* = o.045 in placebo) Follow up: 6 months	New plaque (1 patient from PRP group); hematoma (1 patient from placebo group)	Small sample size; short follow up; high dropout rate; protocol inefficacy

The included studies examining the efficacy of Platelet-rich plasma injections in the treatment of Erectile Dysfunction

*EHS* Erection hardness scale; *ED* Erectile dysfunction; *EDV* End diastolic volume; *IIEF-5* 5 item version of the International Index of Erectile Function questionnaire; *IIEF-EF* Erectile function domain of the international index of erectile function questionnaire; *Li-SWT* Low intensity shock wave; *MCID* Minimal clinically important difference; *PD* Peyronie’s disease; *PDE5i* Phosphodiesterase-5 inhibitor; *PRFM* Platelet-rich fibrin matrix; *PRP* Platelet-rich Plasma; *PSV* Peak systolic volume; *RI* Resistive index; *RPM* Rotations per minute; *VAS* visual analogue scale; *SEP* sexual encounter profile

**Table 2:** Studies investigating Platelet-rich Plasma therapy in men with Peyronie’s Disease

Author	Year	Study design	Study population	Inclusion/ Exclusion Criteria	Groups	PRP preparation method	Treatment method	Results	Adverse effects	Limitations
Virag [29]	2017	Prospective cohort	90 patients (no information on PD stage) Mean age: 56 years (IQR: 25–77)	Inclusion criteria: penile deformation and/or curvature linked to palpable plaques in the tunica albuginea; no previous local treatments Exclusion criteria: not reported	N/A	Autologous PRP with hyaluronic acid; sample was centrifugated for 5 min at 1500 G	Injections with 8 mL solution containing 6 mL PRP and 2 mL hyaluronic acid, with a 15-day interval through a 22G and/or 18G needle. An average of 7.09 injections/patient. Needle fracturing of plaques was performed prior to injecting the solution. All procedures were ultrasound guided. Follow up 3 months after the first injection and every 3rd month afterwards	Mean angel reduction of 16.54 degrees (39.65%). 62.2% reported subjective improvement in degree of curvature. Average improvement of IIEF-5 scores of 4.09 points. Overall, the researcher evaluated that 73.3% had satisfactory results, 12.2% had unsatisfactory results, and 14.4% had bad results	Ecchymosis in 16.7% of cases. Hematoma in 10% of cases. One patient reported worsening of symptoms and were offered surgical treatment	Small sample size. No control groups
Matz [20]	2018	Retrospective cohort study	12 patients (no information on PD state) Mean age: 46 years (IQR: 27–61)	Inclusion criteria: patients affected by PD Exclusion criteria: not reported	N/A	Autologous PRFM (PRP activated by calcium chloride). Sample was centrifugated for 6 min at 6000 rpms	Injections upon patient request; 4-9 mL PRFM pr injection, and average of 2.1 injections/patient after induction of artificial erection with 20 μg of Alprostadil. Three patients received needle fracture of plaques with 10 mL saline prior to PRFM injections. Mean follow up time of 15.5 months	80% reported subjective improvement in degree of curvature. Average improvement of IIEF-5 scores of 4.14 points	Mild pain. Mild penile bruising	Small sample size. No control groups. No objective outcome measurements
Achraf [30]	2022	Prospective cohort study	65 patients Group 1: 33 patients, mean age (SD) 60.4 years (± 10.2) Group 2: 32 patients, mean age (SD) 59.8 years (± 9.9)	Inclusion criteria: stable symptomatic PD; penile curvature between 25°-45°; naïve to treatments; dorsal/lateral/dorsolateral curvature; palpable plaque (> 6 months) Exclusion: Curvature degree < 25° or > 45°; previous treatments for PD; priapism; calcified plaque; not naïve to oral/intralesional treatments	Group 1: Patients with degree curvature 25°-35° Group 2: Patients with degree curvature 35°-45°	Autologous PRP; sample centrifugated at 2750 rpm for 8 min, then 2850 rpm for 8 min more	Injection of 8 mL of PRP intra- and peri-lesion then at the level of tunica albuginea. Injection diagram: injection every 15 days for 2 months, then at 3,6, and 9 months. Patients received an average of 6.1 injections during the study period Follow up time:	Mean curvature reduction of -16.88° (SD 3.35) in group 1 and -17.27° (SD 4.22) in group 2; difference between groups = 0.3° (*p* < 0.001) Improvement in pain measured by VAS: -34% (SD 2.2) in group 1 and -39% (SD 2.9) in group 2 Improvement in IIEF score: + 50% (SD 2.5) in group 1 and + 61% (SD 3.2) in group 2 Increased size of the penis: + 8.1% (SD 2.04) in group 1 and + 7.92% (SD 1.64) in group 2 Follow up at 1, 3, 6, 9 and 12 months	Superficial hematoma at the injection site (5 patients); Mild pain the injection site (8 patients)	No control groups; short follow up; small sample size; no randomization
Chu [32]	2023	Randomized controlled trial, placebo, crossover	Currently enrolled: 28 patients (Target: 80 participants). Group A: 14 patients, mean age (SD) 55.1 (± 9.1) years Group B: 14 patients, mean age (SD) 52.2 (± 11.7) years	Inclusion criteria: men aged 18–75 years with active and chronic PD; palpable plaque; penile curvature 20–120° Exclusion criteria: penile surgery; intralesional injection (< 6 months); priapism; penile fracture; sever ED; hour-glass deformity; ventral plaque	Group A: PRP then saline Group B: saline then PRP	Autologous PRFM (PRP activated by calcium chloride) by Arthrex Angel PRP system	Two injections with 0.5 mL PRFM (group A) or saline (group B) with a two-week interval. Crossover after 3 months. Total follow-up time of 6 months	No difference in curvature at 3-month follow-up (measured by goniometer on artificial erection)	None reported	The study is still ongoing. Small sample size
Zugail [28]	2023	Prospective, non-randomized, cohort study	54 patients median age 47.50 (IQR: 40–55) years	Inclusion criteria: consecutive male patients with PD (difficulties to coitus due to palpable TA plaque) Exclusion criteria: Curative dose of anticoagulant medications; unstable plaques; curvature that has no impact on sexual performance; ED nonresponsive to treatments	N/A	Autologous PRP; sample was centrifugated at 3500 rpms for 5 min	Injection of 5–6 mL of PRP into the plaque after creating multiple channels along the entire longitudinal axis of the plaque through a 25G needle. Each session scheduled 4 weeks apart for a total of 6 sessions Penile vacuum therapy was initiated on day 14 after each session for a daily use of 30 min each day Total follow up time: 7 months after the first injection	Improvement in penile curvature (mean curvature 53.98 ± 23.19° vs 30.09 ± 20.61°; *p* = 0.001)	Ecchymosis at injection site (75.9%); skin infection (1.85%) VAS score was 3 (IQR 0–4.25) at baseline and 2 h after injection 20.37% (11/54) patients referred pain	Small sample size; lack of placebo group; cannot exclude if the results are due to PRP or vacuum; how self-photographs of the penis are taken may affect the outcomes
Alshuaibi [31]	2023	Prospective, non-randomized, cohort study	36 patients, median age 54.08 (± 8.8) years	Inclusion criteria: penile curvature due to palpable stable plaque in TA Exclusion criteria: nonstable plaques, curvature with no impact on the sexual performance, and ED not responding to treatment	N/A	Autologous PRP; sample was centrifugated at 3500 rpms for 5 min	After the creation of multiple holes along the entire longitudinal axis of the plaque through a 25Gx5-8 inches needle, and penile manipulation for 5 min, each patient received the Injection of 10–12 mL of PRP into the plaque on erected penis; additional 5 min of penile manipulation followed. Use of vacuum started on the 7th day Total follow up time: 1 year	The pretreatment mean curvature degree was 57.5 ± 20.61. After the protocol, the mean curvature degree was 40.86 ± 25.13. The mean improvement difference of 16.85 ± 14.81 (*p* = 0.0001),	Haematoma and ecchymosis in 13.88% (5/36) of patients, including one patient who had an “eggplant penis”. No serious adverse incidents were recorded, but two patients had a relapse of curvature after three and four months (5.55%)	Small sample size; lack of a placebo group; follow ups based on photos took by patients; no assessment of the patients’ erectile functions and PD symptoms by Peyronie's Disease Questionnaire (PDQ)

The included studies examining the efficacy of Platelet-rich plasma injections in the treatment of Peyronie’s Disease

*PD* Peyronie’s disease; *PRFM* Platelet-rich fibrin matrix; *PRP* Platelet-rich Plasma

Seventeen studies (four Randomized Controlled Trials [RCT], 11 prospective cohort studies and two retrospective cohort studies including a total of 1099 patients) described the outcomes in patients receiving PRP injection therapy as a therapy for ED or PD. One of the studies included both ED and PD patients. Supplementary Figs. 1–6 illustrate the risk of bias of the included studies, with the majority of studies deemed at high risk of bias.

### PRP and erectile dysfunction

Twelve publications regarding ED were identified, including two retrospective case series, seven prospective uncontrolled trials, and three randomized controlled trials (Table 1). All studies evaluated erectile function improvements using either the 5-item or the erectile function domain of the International Index of Erectile Function questionnaire (IIEF-5 and IIEF-EF, respectively) [14, 15]. Four studies also used penile duplex ultrasound to investigate end-diastolic velocity (EDV), peak systolic velocity (PSV), resistivity index (RI) and mean artery diameter [16–18]. Side effects were generally limited and included mild pain, bruising and development of a small fibrotic plaque in a single patient [19]. No major adverse events were reported in any of the studies.

The first identified study was a retrospective trial published by Matz et al. in 2018 [20]. It included 17 patients, 5 of whom suffered from organic ED. After initial preparation of autologous blood, the authors added a calcium chloride solution, converting fibrinogen to fibrin to create what they termed platelet-rich fibrin matrix. This may bind platelets at the site of injection for a longer time but is still considered a form of PRP. The patients received 1 to 8 injections upon their own request and were followed up at various timepoints. After a mean time of 15.5 months, the IIEF-5 scores improved by an average of 4.14 points (no *p*-value given). However, the completeness of follow-up was unclear, and the visits were guided by the subjective response of the patients likely skewing the results toward an increasing function score. Additionally, there was no mention of concurrent ED treatments, and no absolute IIEF-5 values were mentioned for any time-point. In the second retrospective study, Geyik et al. compared the effectiveness of three PRP injections combined with low-intensity shock wave therapy (Li-SWT) to that of Li-SWT alone in 184 PDE5-I non-responders [21]. All patients were allowed to continue using PDE5-Is and after 6 months the IIEF-EF score increased from 14.33 ± 4.39 to 23.8 ± 4.37 (*p* < 0.001) for the Li-SWT only group, while it increased from 17.82 ± 3.44 to 26.3 ± 2.55 (*p* < 0.001) in the combination group. No statistically significant differences between the groups were reported, suggesting no added effect of PRP. However, these results should be interpreted with caution as the two groups were not comparable regarding baseline characteristics and the selection criteria for choice of treatment were unclear. Further, it is unclear if the study looked at consecutive patients or if some treated men had been lost to follow-up.

In a more structured study, Tas et al. prospectively evaluated 31 treatment naïve ED patients with metabolic syndrome for 6 months following three PRP injections [19]. Here, the median IIEF-EF score increased from 18 to 20. While this change was statistically significant, the low magnitude of change indicated a lack of a clinically meaningful effect. A similar study by Wong et al. (n = 30) found an increase in the mean IIEF-5 score from 12.03 ± 5.10 at baseline to 16.59 ± 5.5 two weeks after the third PRP injection (*p* < 0.001) [22]. While the concurrent use of PDE5-Is in most of the participants represents a potential confounder, an increase of this size does meet the minimal requirement for a clinically relevant change. The prospective interventional cohort study conducted by Sajjad et al. compared the efficacy of Li-SWT and PRP in a cohort of 60 patients affected by ED [23]. Patients were non-randomly assigned to the PRP group, receiving multiple-sites weekly injections for a 6-week-period, or the Li-SWT group, receiving 300 shocks twice a week for a total of 6 weeks. Even though a high percentage of positive results have been reported by both groups, the mean IIEF-5 score improvement at 12-weeks follow up was not statistically significant (*p* > 0.005) [23]. Two more prospective case series were conducted by Zaghloul et al. [17, 18]. In both studies, PDE5-I non-responders were prescribed a daily dose of 5 mg tadalafil and 20 mg vardenafil on demand and subjected to a PRP injections. In the first study (n = 34) the mean IIEF-5 score increased from 7.7 ± 2.7 to 13.2 ± 6.8 (*p* < 0.001) at 3 months follow-up, while no statistically significant changes in penile duplex ultrasound parameters were observed [17]. In the second study an increase in the mean IIEF-5 score from 8.04 ± 2.7 to 12.1 ± 5.6 (*p* = 0.003) was observed in diabetic men (n = 24) and an improvement from 10.2 ± 0.9 to 14.8 ± 4.8 (*p* = 0.001) in non-diabetic men (n = 24) [18]. EDV and PSV also increased at 3 months follow-up. Although these results seem encouraging, it must be stressed that the very high dosing of PDE5-Is is likely to account for at least part of the improvement as it represents a 50% increase from the previous dosing of the participants [18]. A study conducted by Schirmann et al. enrolled 15 patients affected by vascular ED who had not responded to previous treatments [24]. According to the protocol, in each of the three sessions conducted 15 days apart, the patients received a 3 mL injection of PRP combined with sodium citrate in each corpus cavernosum, along with a 6 mL subcutaneous injection. A statistically significant improvement in the IIEF-EF score was observed during follow-ups, with the score rising from 11.80 ± 5.51 at baseline to 16.80 ± 4.97 (*p* = 0.001) at the one-month follow-up, 16.23 ± 5.10 (*p* = 0.003) at the three-month follow-up, and 15.15 ± 6.44 (*p* = 0.02) at the six-month follow-up. The improvement in sexual discomfort score was evident only at the one-month follow-up (6.67 ± 19.97 vs. 16.67 ± 24.40; *p* = 0.043). However, it is noteworthy that the EHS and SEP did not exhibit improvement following the treatment. [24].

The prospective non-randomized cohort study conducted by Francomano et al. aimed to assess the response to PDE5-Is before and after PRP injection in a cohort of 150 vasculogenic ED patients [25]. A 5 mL PRP solution was injected into two sites in each corpora cavernosa, and no serious adverse events were reported. At the 1-month follow-up, nearly all patients (80%) resumed sexual activity and reported an improvement in the IIEF-5 score after using PDE5-Is, along with enhanced cavernosal blood flow following pharmacological stimulation. The results showed a significant increase in IIEF-5 scores from 12 ± 2.6 at baseline to 19 ± 3.0 after treatment (*p* < 0.0001) and a rise in PSV from 32 ± 5.5 cm/s before treatment to 42 ± 7.6 cm/s after treatment (*p* < 0.0001). Additionally, the study proposed that mean platelet volume (MPV) at baseline could serve as a predictive biomarker for PRP treatment outcomes, with lower MPV values associated with a higher likelihood of treatment response [25]. The final studies on ED and PRP are randomized, double-blind, placebo-controlled trials. Poulios et al. randomized men with mild or moderate vasculogenic ED to receive two PRP (n = 30) or placebo (n = 30) injections [26]. The effect was evaluated based on a minimal clinically important difference (MCID) defined as an improvement in the IIEF-EF score by 2 or more points for mild or mild to moderate ED or 5 or more points for moderate ED. At 1-, 3-, and 6-months follow-up, 22/29 (76%), 20/29 (69%), and 20/29 (69%) patients in the PRP group achieved the MCID, respectively. The corresponding numbers in the placebo group was 7/28 (25%), 10/26 (39%), and 7/26 (27%) with statistically significant between-group differences at all time points. In the second randomized trial, Shaher et al. also divided men with vasculogenic ED between PRP (n = 50) and placebo (n = 50) groups, using the same MCID definition as Poulios et al. [16, 26]. At 1-, 3-, and 6-months follow-up, 38/50 (76%), 36/50 (72%), and 35/50 (70%) patients in the PRP group achieved the MCID, compared to 9/50 (18%), 8/50 (16%), and 8/50 (16%) in the placebo group (*p* < 0.001 for all time points). The authors also observed improvements in penile blood flow on duplex ultrasound in the PRP group. While these studies do indicate a possible short-term effect of PRP, it is important to highlight that both studies find relatively small mean changes in the IIEF-EF scores of treated patients (3.3 and 4 points, respectively) and a surprising lack of overall placebo effects. Additionally, only the Poulios trial appears well designed with clear description of the blinding and randomization procedures and prohibition of PDE5-I use throughout the study. Thus, the study by Shaher et al. is severely hampered by methodological drawbacks as it makes no mention of concurrent ED treatments, fails to perform any statistical comparisons between the overall IIEF-EF scores in the PRP and placebo groups, and was not pre-registered in any form. In the third randomized controlled trial, Masterson et al. allocated a cohort of organic ED patients into PRP (n = 28) and saline solution (n = 33) groups [27]. The authors employed the same MCID definition used in the two aforementioned studies [16, 26]. At the 1-month follow-up, 58.3% (14/24) in the PRP group vs. 53.6% (15/28) in the placebo group achieved MCID (*p* = 0.730). Statistically significant changes in the mean IIEF-EF score from baseline were observed in both groups at 1 month (mean increase of 3.7 vs. 3.1; *p* = 0.026 vs. *p* = 0.009) and at 6 months (mean increase of 5 vs. 2.2; *p* = 0.003 vs. *p* = 0.045), but no statistically significant differences were noted between the two groups (*p* = 0.765 and *p* = 0.116). The increase in IIEF-EF at 3 months was not statistically significant, and there was no significant difference between the two subsets (*p* = 0.662). Despite efforts to reduce methodological drawbacks and bias through a double-blinded design, several limitations were acknowledged, including a small sample size, a high dropout rate, a short follow-up time, and differences in patient populations; furthermore, the authors reported a higher prevalence of patients with prediabetes in the control group [27].

### PRP and Peyronie’s disease

The literature search revealed four prospective cohort studies, one retrospective case series (also included for ED), and a publication with preliminary results from a placebo controlled randomized trial examining PRP as a therapeutic intervention for PD (Table 2). The PRP injections were administered intralesional in all studies and side effects were generally minor and self-limiting including bruising, hematoma, ecchymosis, and mild pain. Only one case of skin infection was reported during a trial creating multiple channels along the entire longitudinal axis of the plaque [28]. Across the studies a single patient was reported to discontinue PRP treatment after four injections due to PD aggravation [29].

The first study was published by Virag et al. in 2017 [29]. This was a prospective cohort study investigating a combined injection of PRP and hyaluronic acid in 90 participants with established penile plaques and deformity. The authors used initial plaque needle fracturing and subsequent injections were performed upon patient request, with a reported average of 7.09 injections per patient. After the treatments a mean angle reduction of 16.54 degrees corresponding to a ~ 40% reduction in curvature (mean curvature before injection of 44.37 degrees ± 15.93 degrees) was observed. A potential issue in this regard is that the maximum curvature was reportedly measured on photographs of fully erected penises although approximately 1/3 of the participants in the study had ED. The objective improvements were accompanied by statistically significant reductions across the three domains of the Peyronie’s Disease Questionnaire and on subjective assessment 67.8% felt that the treatment had improved their initial condition.

In another prospective cohort study, conducted by Achraf et al., 65 patients affected by stable symptomatic Peyronie's Disease (PD) were enrolled [30]. These patients had not undergone any previous PD treatments. The cohort was subsequently divided into two groups based on the degree of penile curvature: 25°–35° in group 1 and 35°–45° in group 2. Each patient underwent 8 mL of PRP at each session, with an average of 6.1 injections in total. The findings revealed a significant improvement in penile curvature, reduction in pain, and enhanced erectile function following PRP injections. Specifically, a mean curvature reduction of 16.88° and 17.27° was noted in groups 1 and 2, respectively. Pain during sexual intercourse decreased (VAS: -34% in group 1 and -39% in group 2), and erectile function showed improvement as assessed by the IIEF questionnaire (+ 50% in group 1 vs. + 61% in group 2) [30]. The prospective cohort study published in 2023 by Zugail et al., enrolled 54 patients affected by stable PD who reported difficulties to perform coitus due to the disease [28]. The main procedure involved percutaneous needle tunnelling by creating multiple channels along the longitudinal axis of the plaque; then, 5–6 mL of PRP were injected, and 14 days after, daily use of vacuum device was initiated. Despite promising results being reported, with an improvement in penile curvature from 53.98 ± 23.19° at baseline to 30.09 ± 20.61° post-treatment (*p* = 0.001), it is impossible to establish if the benefits came from PRP injections or vacuum therapy. Analogously Alshuaibi et al. also applied a combined treatment approach using percutaneous needle tunnelling followed by penile modeling and PRP injection [28, 31]. However, in contrast to the aforementioned protocols, patients received the treatment after artificially induced erections and during general anesthesia; these measures could ensure a more accurate assessment of penile curvature and plaques position. Results from the cohort of 36 patients suggest a statistically significant improvement in penile curvature, with a mean improvement difference of 16.85 ± 14.81 (pretreatment mean curvature 57.5 ± 20.61 vs 40.86 ± 25.13 after treatment; *p* = 0.0001). Unfortunately, these promising outcomes are limited by assessment of penile curvature improvement by photographs taken by the patients and the lack of validated questionnaires for ED and PD evaluation. The retrospective case series was the one by Matz et al. described previously, in which the authors used a version of PRP termed platelet-rich fibrin matrix [20]. Twelve of the patients enrolled in this study suffered from PD and three of them underwent needle fracture of their plaques prior to PRP injections. However, no further details regarding the patients were reported and the paper simply states that 4/5 patients with available follow-up data reported subjective curvature improvement. The last paper on PRP and PD presents preliminary data from a randomized placebo-controlled crossover trial with an inclusion target of 80 men [32]. In this trial participants are randomized to either two intralesional PRP injections or two saline injections with planned cross over after 3 months. At the time of publication, 3 months follow-up data were available for 17 men and showed no changes in penile curvature in either group compared to baseline.

## Discussion

No standardized PRP preparation methods or treatment protocols exist, and the treatment is in its infancy in the field of andrology. This makes it difficult to compare results across studies and to make definite conclusions regarding efficacy. Specifically there is a lack of a defined protocol for PRP preparation, agreement on injections timing or even the dose to administer and PRP therapy is not recommended by neither the EAU nor AUA guidelines. Thus, the treatment still represents an off-label therapy, and it is mainly applied in clinical trials. However, PRP is already being promoted as a curative treatment for sexual dysfunctions and is being offered to patients [6]. Therefore, it is important for clinicians dealing with these issues to be aware of the available data.

The main strength of our review is the inclusion of all clinical studies on PRP in the treatment of ED and PD including in dept descriptions of individual trials. However, the number of original studies is limited, and the individual trials contain important drawbacks, which limits our ability to draw firm conclusions. Overall, the literature on PRP is scarce with only few studies identified. The trials consistently show that the treatment is safe and no patients in any of the studies have experienced severe side effects after injections. The studies we selected are challenging to compare due to the heterogeneity of the cohort analyzed, as well as the barely defined inclusion and exclusion criteria. Indeed, studies enrolling patients affected by ED generally focus on vasculogenic or organic ED when specified. However, in some cases, exclusion criteria are not very restrictive, and iatrogenic ED patients are included as well [25]. Only one of the studies enrolling patients affected by PD specified the disease status [30]. In several studies positive effects of the treatment were reported and the use of validated questionnaires and scales provides some merit to these findings. However, most of the clinical trials are limited by their observational designs, small sample sizes, and short duration of follow-up. Notably, only four studies were randomized [16, 26, 27, 32] and two were case–control studies [21, 23]. In some cases, there was even a lack of a standardized treatment protocol within the individual study. The lack of control groups is especially problematic in conditions such as ED and PD because of the important psychological components when dealing with sexual dysfunction. Thus, it is well documented that placebo medications have a measurable effect in ED patients and there are several plausible biological explanations to explain this including changes in arousal and activation of central dopaminergic pathways [33]. Likewise, well designed clinical studies in PD patients have shown placebo effects on both objective and subjective parameters and bother from the condition may subside over time in some men even without treatment [34, 35].

For ED, we only identified one well designed and adequately reported randomized controlled trial [26]. This study showed benefits of PRP compared to placebo injections with potentially clinically relevant responses in a total of 20 men. However, the study definition of a MCID for the IIEF-EF questionnaire of 2 points in men with mild to moderate ED is questionable since this group was actually not specifically described when MCID values were determined for the questionnaire [36]. As this sub-group of men comprised most study participants it seems reasonable to remain somewhat skeptical of the clinical benefits. This is of particular importance as the established overall MCID for IIEF-EF is 4 points, while the study found a mean improvement of 3.3 points. Finally, it warrants mentioned that similar improvements have previously been noted in purely placebo treated patients and that the magnitude of the improvements is approximately 10 points on the IIEF-EF for PDE5-Is [37, 38].

For PD, no controlled studies were available and only one completed trial and two prospective cohort studies provided objective curvature measurements [28–30]. In the trial, the combination of PRP and hyaluronic acid injections looked promising with an improvement of a similar magnitude as what has been reported for collagenase injections [34]. However, although very preliminary, the early results from the ongoing randomized trial hints that the benefits may not be reproducible under controlled conditions. Additionally, formal comparisons of outcomes proved challenging due to the variety in follow-up length, typically between 1 and 12 months, as well as the diverse doses and number of PRP injections, varying from 0.5 to 9 mL and from 2 to 6 injections. The lack of agreement also applies to the preparation methods. In fact, the methods for preparing PRP differ in the inclusion of other substances such as calcium chloride, activation factors, and hyaluronic acid, as well as in centrifugation time and number of spins. As previously reported in studies, the concentration of growth factors in PRP preparation seems to vary among patients with ED. Therefore, this might represent an additional complication when comparing results between different studies and patients [39].

## Future directions

One of the primary concerns when evaluating the advantages of PRP is establishing a validated and widely recognized preparation protocol. Standardization is crucial for determining the right dosage, the frequency of injections, the injection locations, potential dilutions with other substances, the number of centrifuge spins, and the duration of centrifugation. Although numerous PRP preparation protocols exist, each asserting its superiority, it would be prudent to standardize individual protocols, considering factors such as cost-effectiveness and their suitability for clinical settings [40]. The matter is further complicated by the finding that PRP growth factors may vary among men [39]. Theoretically this could mean that men with more severe disease may benefit less from PRP but further studies are needed to clarify this. Regarding potential future applications in the andrology field, PRP has not yet been extensively investigated in clinical trials related to male infertility. Nevertheless, in vitro studies have demonstrated encouraging outcomes, showing promising effects of PRP on semen quality and reducing oxidative stress. [41]. It has the potential to facilitate the proliferation of spermatogonial stem cells (SSC) and offer protection to semen samples during cryopreservation. [42–44]. Further trials are needed to assess if this will translate into clinical benefits.

## Conclusion

The existing literature on PRP therapy in andrology remains limited. Trials confirm the procedure's safety, noting only minor and temporary adverse events and most seem to indicate small to moderate positive effects on outcomes for both ED and PD. Nevertheless, caution is warranted when interpreting these findings. Limitations include variations in PRP protocols and several methodological drawbacks. Future research is required to determine the optimal preparation and treatment protocols for PRP therapy, as well as to clarify its effectiveness in andrology.

## Supplementary Information

Below is the link to the electronic supplementary material.

Supplementary file1 (PPTX 617 KB)

Supplementary file2 (DOCX 15 KB)

## Data Availability

All data used in the study are avaliable in the original published trials referenced in the paper.

## Declarations

### Human or animal participants

This systematic review does not involve any human participants or animals. Therefore, ethical approval or animal care guidelines were not required for this study.
