# Supplementary material for: Platelet-rich plasma therapy in erectile dysfunction and Peyronie’s disease: a systematic review of the literature
Source: World J Urol. 2024 May 29;42(1):359. doi: 10.1007/s00345-024-05065-3 (PMC11136842; doi:10.1007/s00345-024-05065-3)
Supplement: Supplementary file 1 — Supplementary file1 (PPTX 617 KB) [file 345_2024_5065_MOESM1_ESM.pptx]

## Slide 1
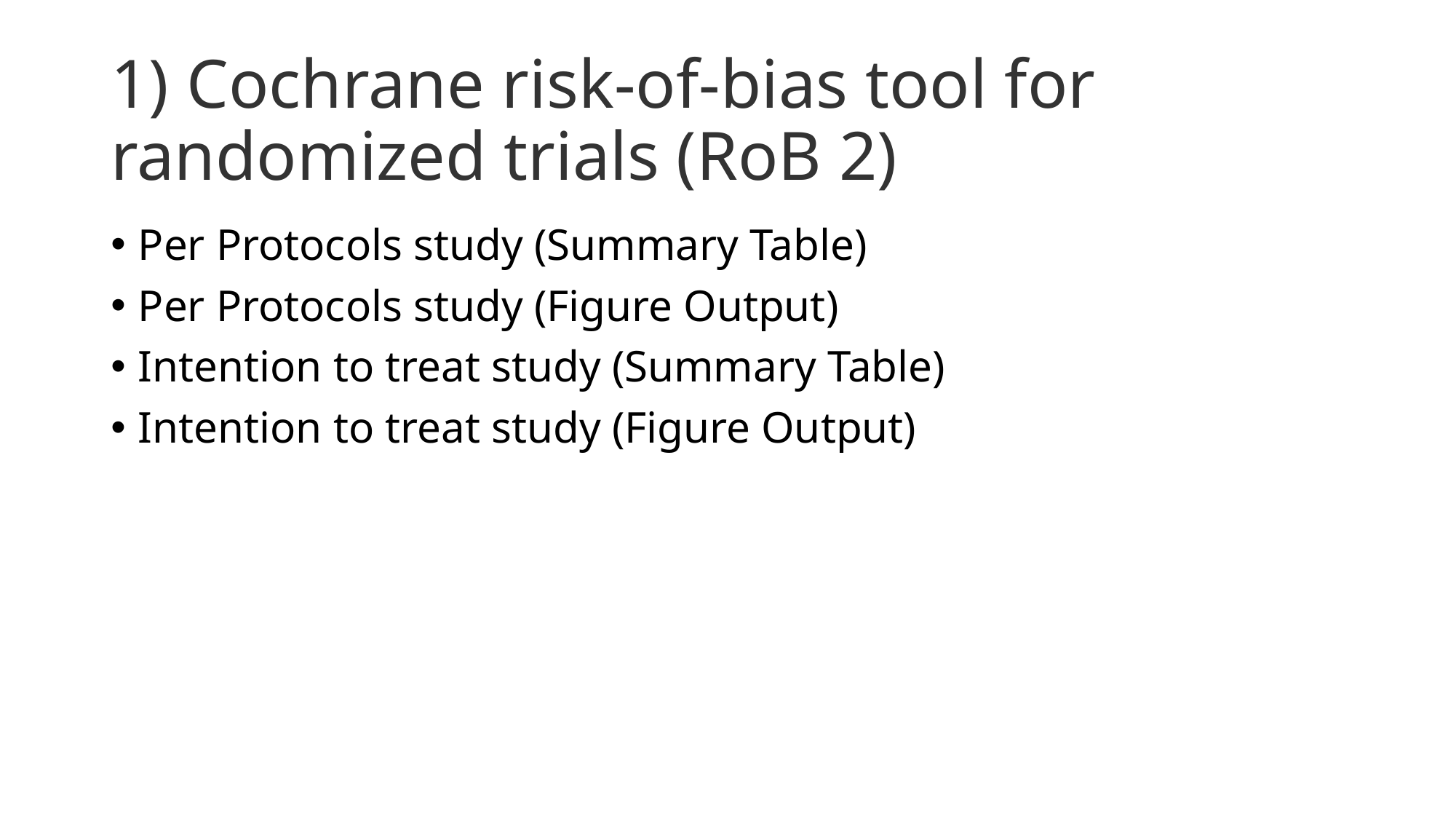

# 1) Cochrane risk-of-bias tool for randomized trials (RoB 2)
Per Protocols study (Summary Table)
Per Protocols study (Figure Output)
Intention to treat study (Summary Table)
Intention to treat study (Figure Output)

## Slide 2
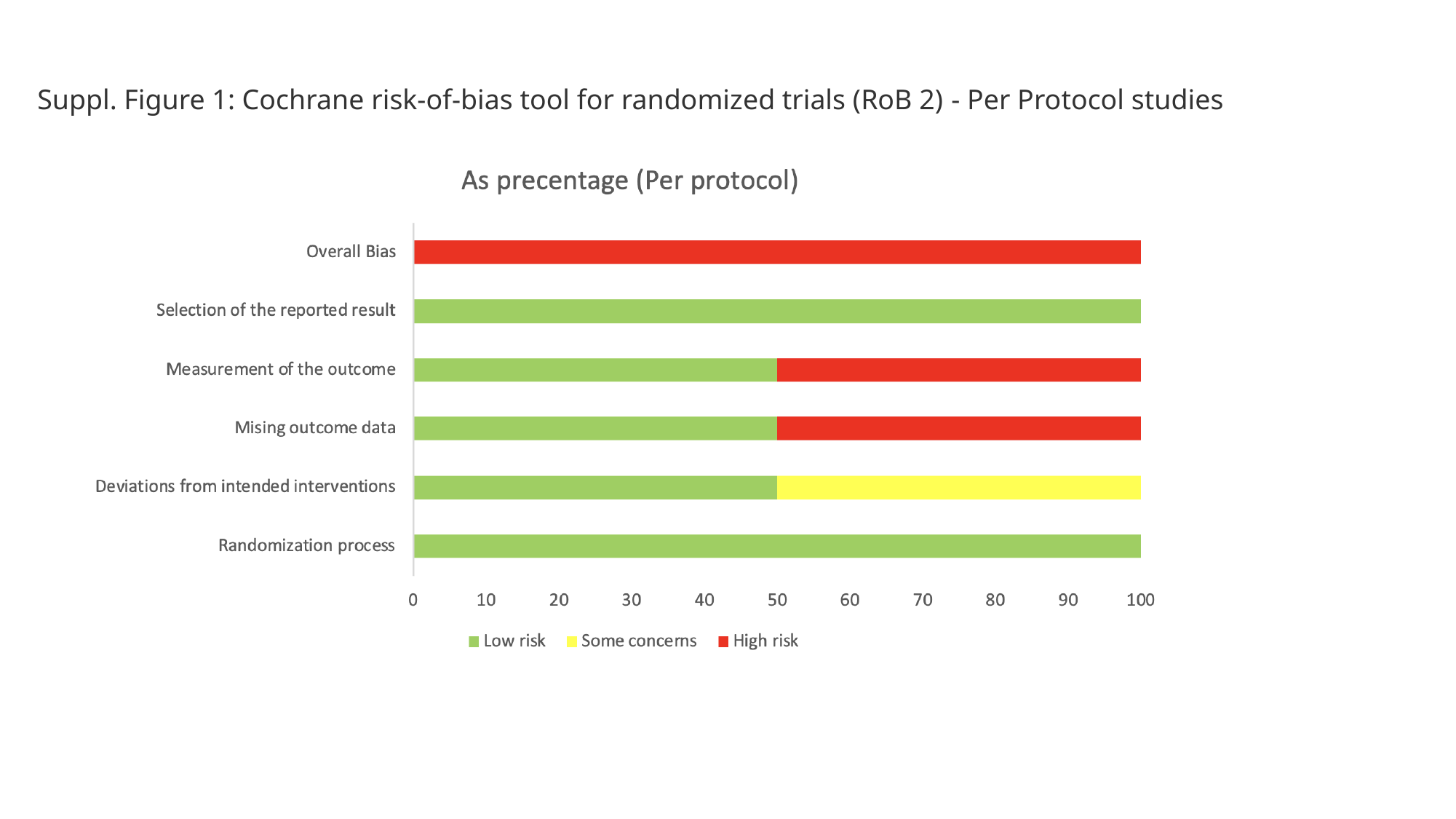

Suppl. Figure 1: Cochrane risk-of-bias tool for randomized trials (RoB 2) - Per Protocol studies

## Slide 3
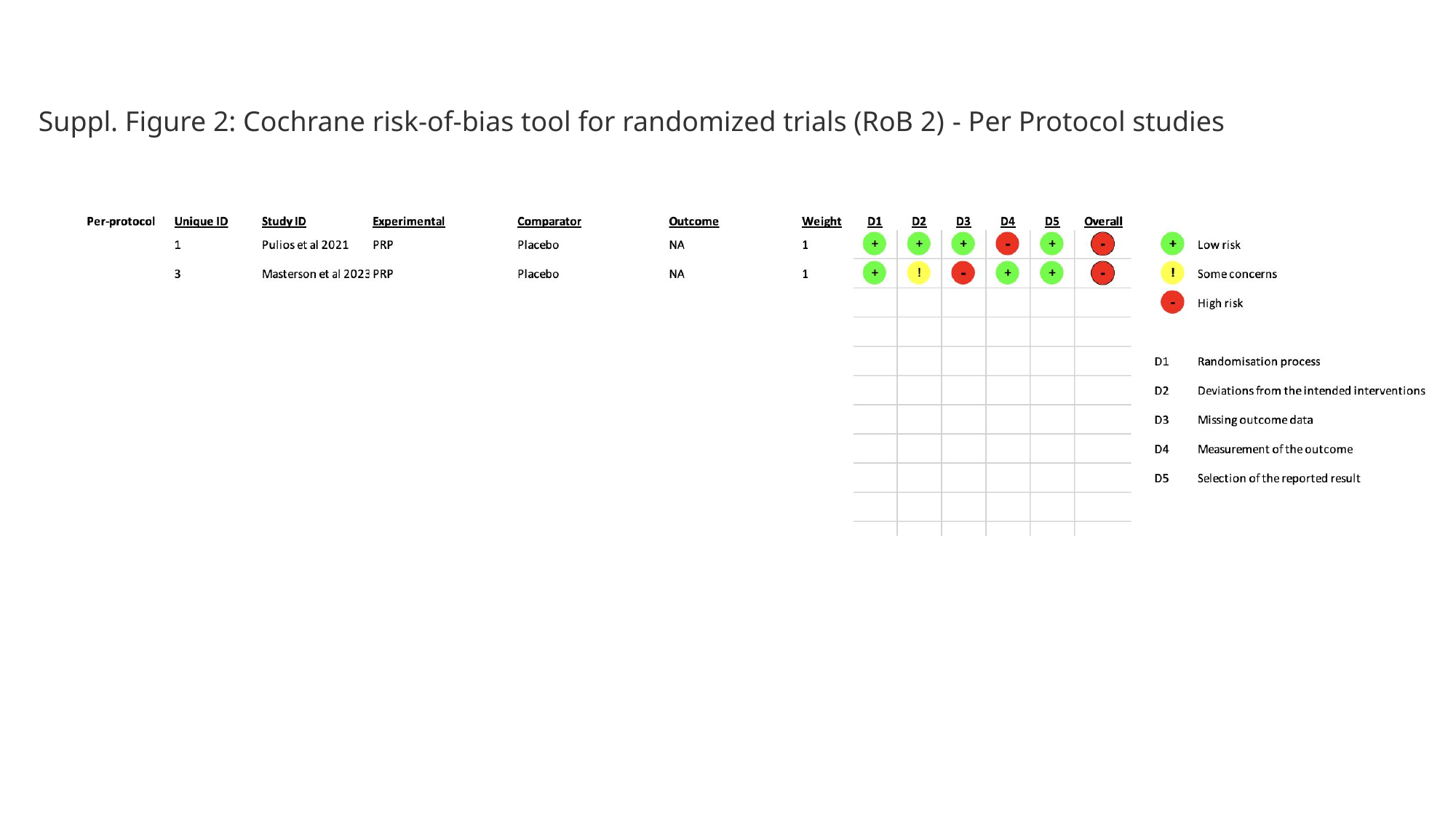

Suppl. Figure 2: Cochrane risk-of-bias tool for randomized trials (RoB 2) - Per Protocol studies

## Slide 4
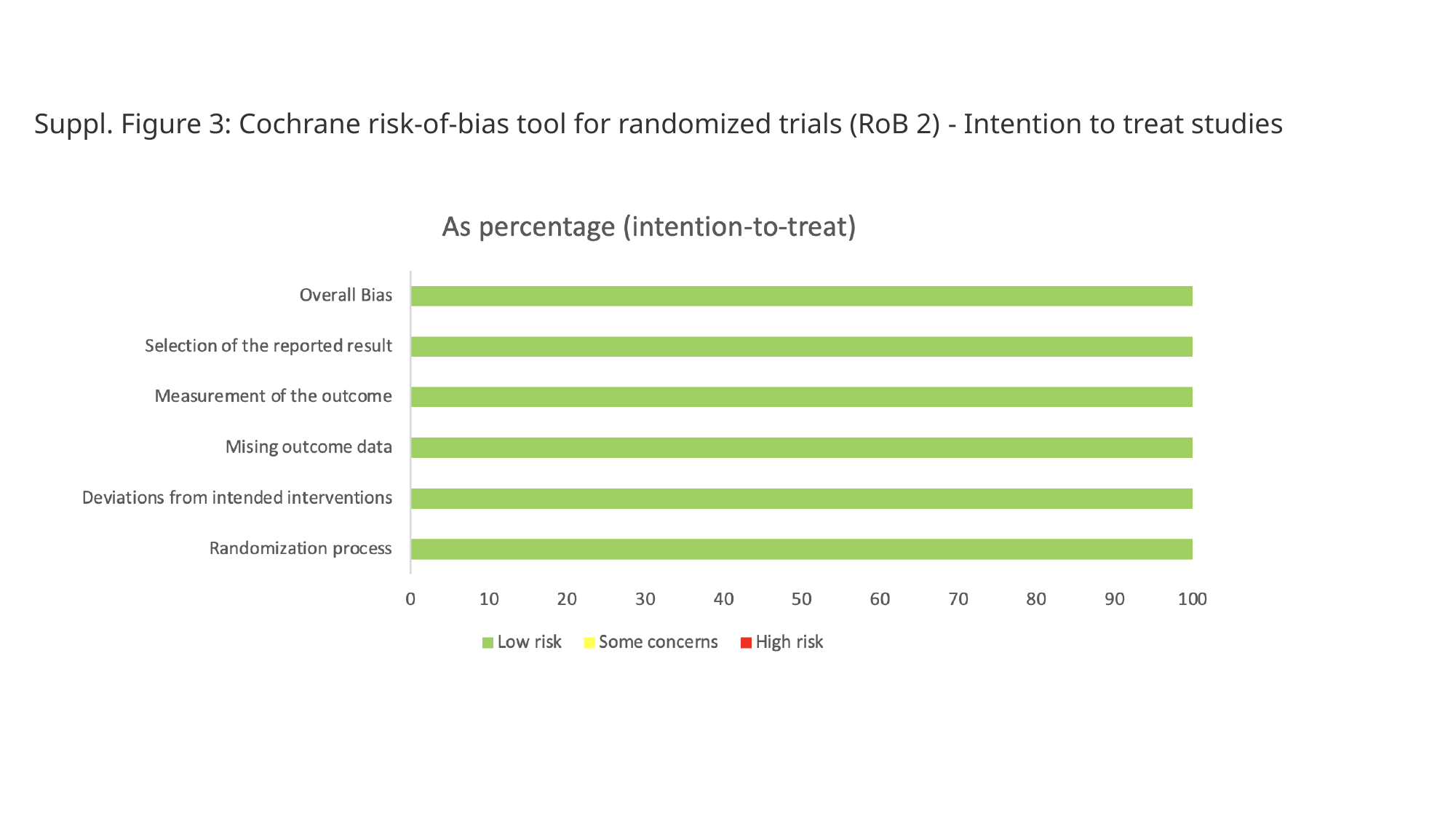

Suppl. Figure 3: Cochrane risk-of-bias tool for randomized trials (RoB 2) - Intention to treat studies

## Slide 5
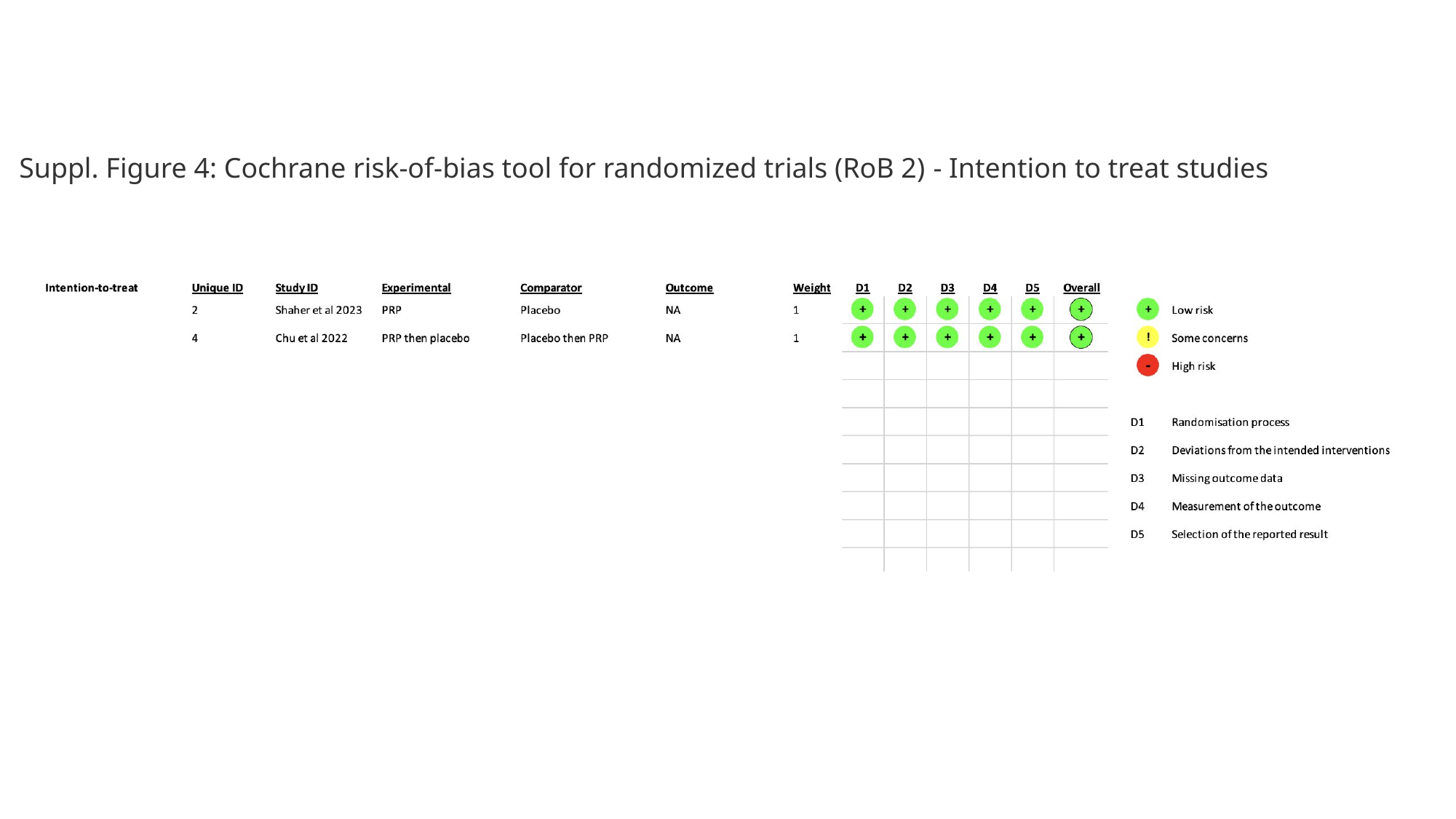

Suppl. Figure 4: Cochrane risk-of-bias tool for randomized trials (RoB 2) - Intention to treat studies

## Slide 6
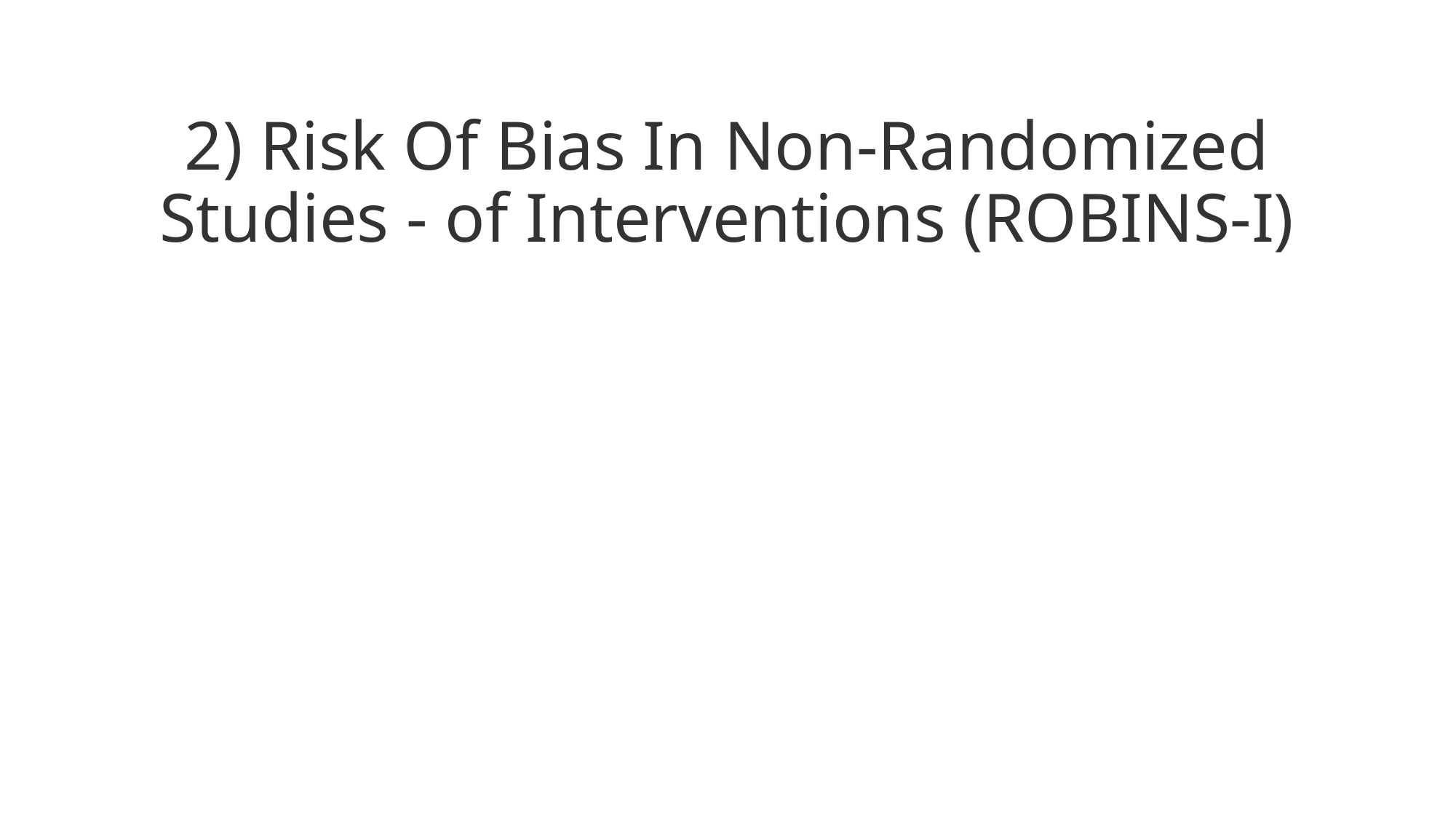

# 2) Risk Of Bias In Non-Randomized Studies - of Interventions (ROBINS-I)

## Slide 7
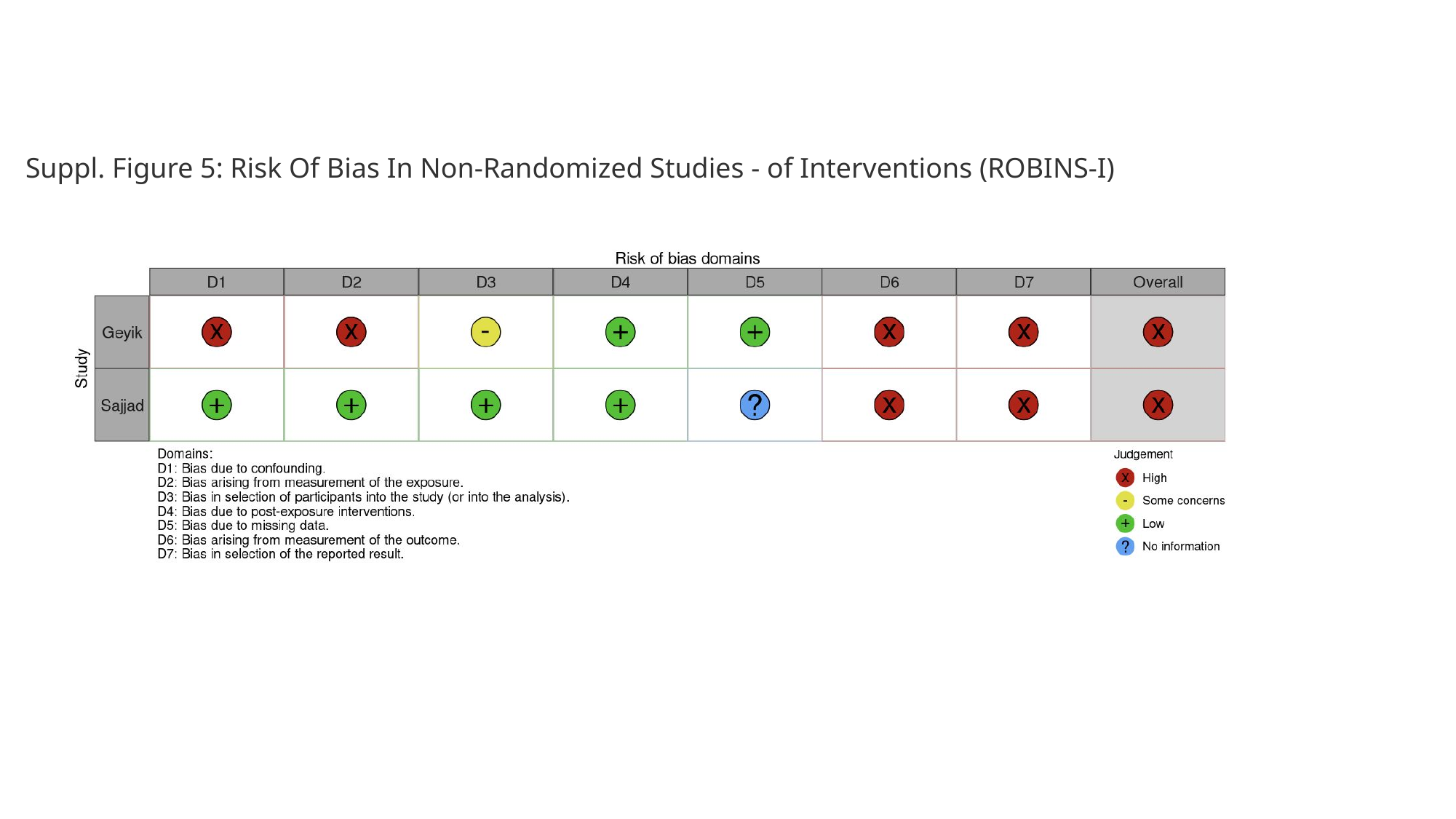

Suppl. Figure 5: Risk Of Bias In Non-Randomized Studies - of Interventions (ROBINS-I)

## Slide 8
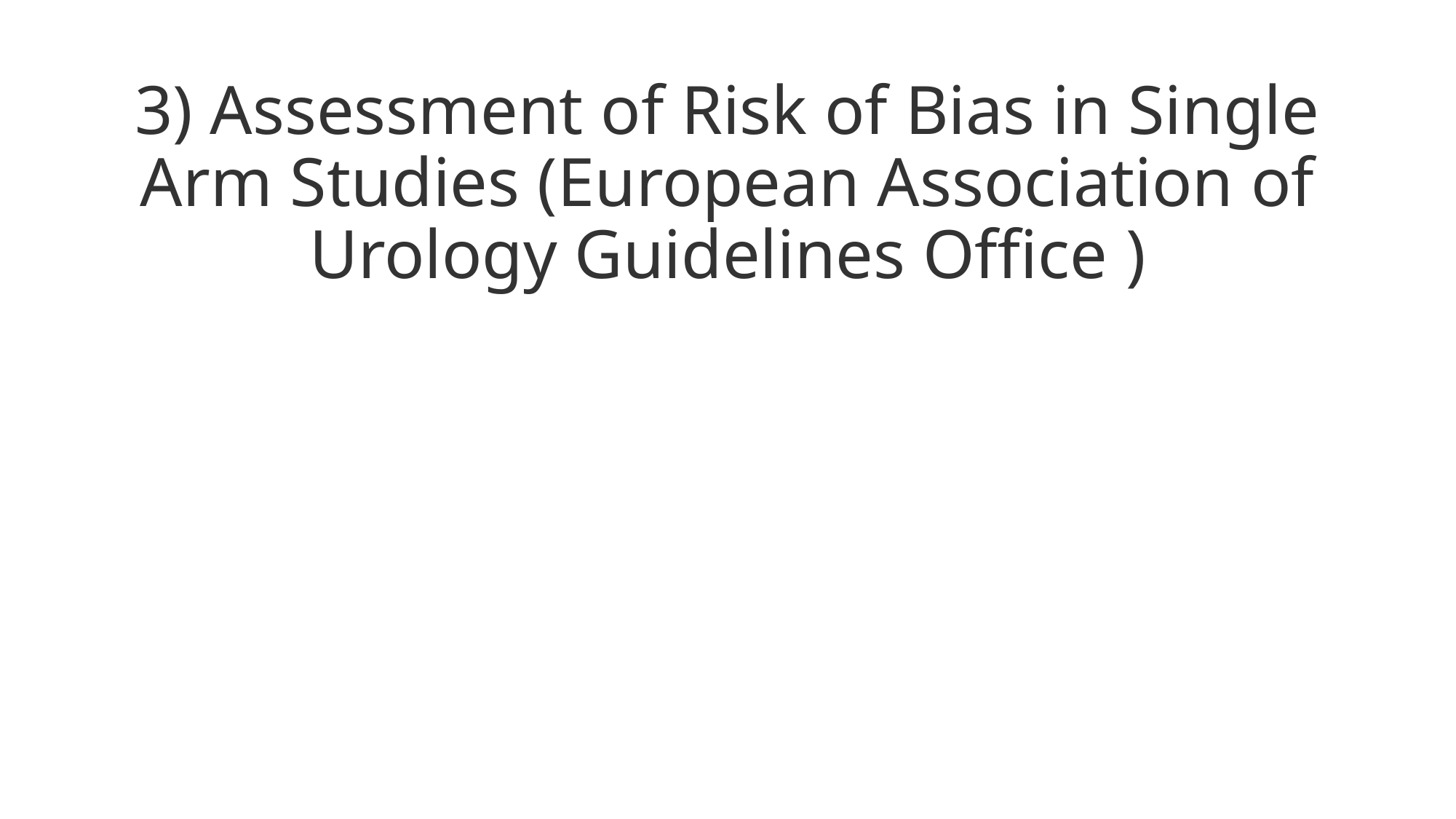

# 3) Assessment of Risk of Bias in Single Arm Studies (European Association of Urology Guidelines Office )

## Slide 9
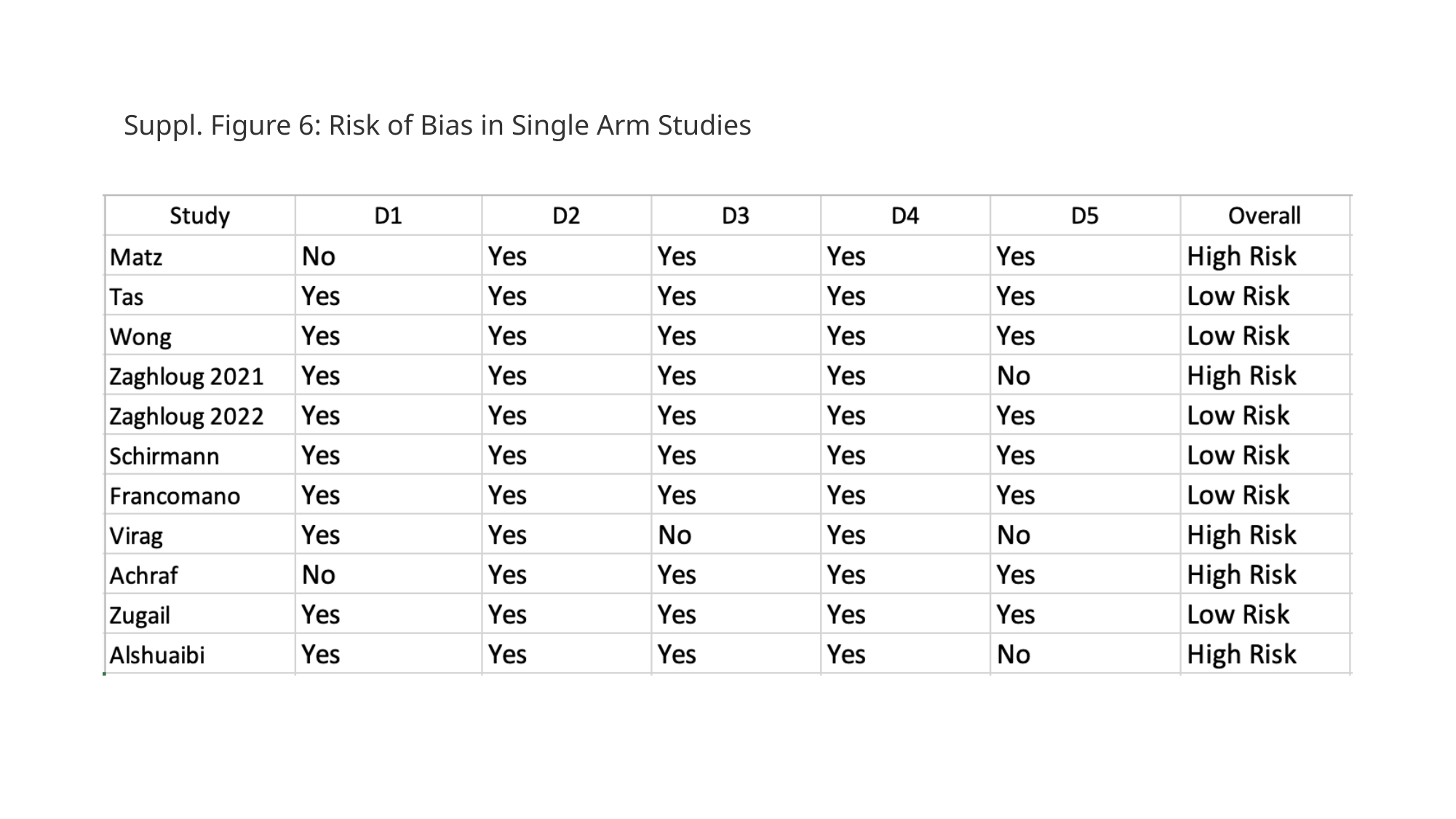

Suppl. Figure 6: Risk of Bias in Single Arm Studies
