# Supplementary material for: Platelet-rich plasma therapy in erectile dysfunction and Peyronie’s disease: a systematic review of the literature
Source: World J Urol. 2024 May 29;42(1):359. doi: 10.1007/s00345-024-05065-3 (PMC11136842; doi:10.1007/s00345-024-05065-3)
Supplement: Supplementary file 2 — Supplementary file2 (DOCX 15 KB) [file 345_2024_5065_MOESM2_ESM.docx]

**Suppl. Table 1.** Population, Intervention, Comparator, and Outcomes (PICO) model of the current study

| **Category** | **Description** |
| --- | --- |
| Population | Human patients affected by ED or PD |
| Intervention | Autologous PRP injection |
| Comparator | Human patients affected by ED or PD receiving other types of treatments (saline solution injection; oral daily/on demand dose of tadalafil/vardenafil; Li-SWT) or no treatments at all |
| Outcomes | - - Evaluation of improved erectile function (IIEF, IIEF5; IIEF-EF) and of degree of curvature   - ED duration, Erection Hardness Score [EHS], end-diastolic velocity [EDV], peak systolic velocity [PSV], resistive index [RI] or arterial diameter |
